# Demonstration of event position reconstruction based on diffusion in the NEXT-white detector

**DOI:** 10.1140/epjc/s10052-024-12865-9

**Published:** 2024-05-21

**Authors:** J. Haefner, K. E. Navarro, R. Guenette, B. J. P. Jones, A. Tripathi, C. Adams, H. Almazán, V. Álvarez, B. Aparicio, A. I. Aranburu, L. Arazi, I. J. Arnquist, F. Auria-Luna, S. Ayet, C. D. R. Azevedo, K. Bailey, F. Ballester, M. del Barrio-Torregrosa, A. Bayo, J. M. Benlloch-Rodríguez, F. I. G. M. Borges, A. Brodolin, N. Byrnes, S. Cárcel, J. V. Carrión, S. Cebrián, E. Church, L. Cid, C. A. N. Conde, T. Contreras, F. P. Cossío, E. Dey, G. Díaz, T. Dickel, M. Elorza, J. Escada, R. Esteve, R. Felkai, L. M. P. Fernandes, P. Ferrario, A. L. Ferreira, F. W. Foss, E. D. C. Freitas, Z. Freixa, J. Generowicz, A. Goldschmidt, J. J. Gómez-Cadenas, R. González, J. Grocott, K. Hafidi, J. Hauptman, C. A. O. Henriques, J. A. Hernando Morata, P. Herrero-Gómez, V. Herrero, C. Hervés Carrete, Y. Ifergan, L. Labarga, L. Larizgoitia, A. Larumbe, P. Lebrun, F. Lopez, N. López-March, R. Madigan, R. D. P. Mano, A. P. Marques, J. Martín-Albo, G. Martínez-Lema, M. Martínez-Vara, Z. E. Meziani, R. L. Miller, K. Mistry, J. Molina-Canteras, F. Monrabal, C. M. B. Monteiro, F. J. Mora, J. Muñoz Vidal, P. Novella, A. Nuñez, D. R. Nygren, E. Oblak, J. Palacio, B. Palmeiro, A. Para, I. Parmaksiz, J. Pelegrin, M. Pérez Maneiro, M. Querol, A. B. Redwine, J. Renner, I. Rivilla, J. Rodríguez, C. Rogero, L. Rogers, B. Romeo, C. Romo-Luque, F. P. Santos, J. M. F. dos Santos, I. Shomroni, A. Simón, S. R. Soleti, M. Sorel, J. Soto-Oton, J. M. R. Teixeira, J. F. Toledo, J. Torrent, A. Trettin, A. Usón, J. F. C. A. Veloso, J. Waiton, J. T. White

**Affiliations:** 1 Department of Physics, Harvard University, Cambridge, 02138 MA USA; 2 Department of Physics, University of Texas at Arlington, Arlington, 76019 TX USA; 3 Department of Physics and Astronomy, Manchester University, Manchester, M13 9PL UK; 4 Argonne National Laboratory, Argonne, IL 60439 USA; 5 Instituto de Instrumentación para Imagen Molecular (I3M), Centro Mixto CSIC-Universitat Politècnica de València, Camino de Vera s/n, 46022 Valencia, Spain; 6 Department of Organic Chemistry I, University of the Basque Country (UPV/EHU), Centro de Innovación en Química Avanzada (ORFEO-CINQA), 20018 San Sebastián/Donostia, Spain; 7 Department of Applied Chemistry, Universidad del Pais Vasco (UPV/EHU), Manuel de Lardizabal 3, 20018 San Sebastián/Donostia, Spain; 8 Unit of Nuclear Engineering, Faculty of Engineering Sciences, Ben-Gurion University of the Negev, P.O.B. 653, Beer-Sheva, 8410501 Israel; 9 Pacific Northwest National Laboratory (PNNL), Richland, WA 99352 USA; 10 II. Physikalisches Institut, Justus-Liebig-Universitat Giessen, Giessen, Germany; 11 Institute of Nanostructures, Nanomodelling and Nanofabrication (i3N), Universidade de Aveiro, Campus de Santiago, 3810-193 Aveiro, Portugal; 12 Donostia International Physics Center, BERC Basque Excellence Research Centre, Manuel de Lardizabal 4, 20018 San Sebastián/Donostia, Spain; 13 Laboratorio Subterráneo de Canfranc, Paseo de los Ayerbe s/n, Canfranc Estación, 22880 Spain; 14 LIP, Department of Physics, University of Coimbra, 3004-516 Coimbra, Portugal; 15 Centro de Física de Materiales (CFM), CSIC and Universidad del Pais Vasco (UPV/EHU), Manuel de Lardizabal 5, 20018 San Sebastián/Donostia, Spain; 16 Instituto de Física Corpuscular (IFIC), CSIC and Universitat de València, Calle Catedrático José Beltrán, 2, 46980 Paterna, Spain; 17 Centro de Astropartículas y Física de Altas Energías (CAPA), Universidad de Zaragoza, Calle Pedro Cerbuna, 12, 50009 Zaragoza, Spain; 18 Instituto Gallego de Física de Altas Energías, Univ. de Santiago de Compostela, Campus sur, Rúa Xosé María Suárez Núñez, s/n, 15782 Santiago de Compostela, Spain; 19 LIBPhys, Physics Department, University of Coimbra, Rua Larga, 3004-516 Coimbra, Portugal; 20 Ikerbasque (Basque Foundation for Science), 48009 Bilbao, Spain; 21 Department of Chemistry and Biochemistry, University of Texas at Arlington, Arlington, TX 76019 USA; 22 Lawrence Berkeley National Laboratory (LBNL), 1 Cyclotron Road, Berkeley, CA 94720 USA; 23 Department of Physics and Astronomy, Iowa State University, Ames, IA 50011-3160 USA; 24 Hebrew University, Edmond J. Safra Campus, Jerusalem, 9190401 Israel; 25 Departamento de Física Teórica, Universidad Autónoma de Madrid, Campus de Cantoblanco, 28049 Madrid, Spain; 26 Fermi National Accelerator Laboratory, Batavia, IL 60510 USA; 27 Escola Politècnica Superior, Universitat de Girona, Av. Montilivi, s/n, 17071 Girona, Spain; 28 Department of Physics and Astronomy, Texas A &M University, College Station, TX 77843-4242 USA; 29 Present Address: Weizmann Institute of Science, Rehovot, Israel

## Abstract

Noble element time projection chambers are a leading technology for rare event detection in physics, such as for dark matter and neutrinoless double beta decay searches. Time projection chambers typically assign event position in the drift direction using the relative timing of prompt scintillation and delayed charge collection signals, allowing for reconstruction of an absolute position in the drift direction. In this paper, alternate methods for assigning event drift distance via quantification of electron diffusion in a pure high pressure xenon gas time projection chamber are explored. Data from the NEXT-White detector demonstrate the ability to achieve good position assignment accuracy for both high- and low-energy events. Using point-like energy deposits from ^83m^Kr calibration electron captures ($E\sim 45$ keV), the position of origin of low-energy events is determined to 2 cm precision with bias $< 1~$mm. A convolutional neural network approach is then used to quantify diffusion for longer tracks ($E\ge ~1.5$ MeV), from radiogenic electrons, yielding a precision of 3 cm on the event barycenter. The precision achieved with these methods indicates the feasibility energy calibrations of better than 1% FWHM at Q_ββ_ in pure xenon, as well as the potential for event fiducialization in large future detectors using an alternate method that does not rely on primary scintillation.

## Introduction

Noble element time projection chambers (TPCs) in the liquid or gaseous phase are a widely used technology for rare event searches. These include the NEXT [1], EXO/nEXO [2, 3], and PandaX [4] experiments for neutrinoless double beta decay ($0 \nu \beta \beta$) searches, and the XENON [5], LUX-ZEPLIN [6], and DarkSide [7] experiments for dark matter searches, among others. The basic operating principle of the TPC is that when a particle interacts in the detector, it produces a flash of light through primary scintillation (S1), and ionization electrons along the path of the particle. Using uniform electric fields applied across the detector volume, the ionization electrons are drifted with a known velocity and collected by a readout system. In electroluminescent TPCs such as NEXT, charge is detected by driving the ionization electrons across a high voltage gap, called an electroluminescence region, in order to produce an amplified secondary scintillation signal (S2). For NEXT-White, the total drift length is 664.5 mm and the drift velocity is 0.91 mm/$\upmu$s. The time difference between the S1 and S2 signals allows the determination of the position in the drift direction *z*, given a known drift velocity. Thus full event reconstruction including absolute placement in *z* requires both S1 and S2 signals to be employed.

Information about *z* is in principle accessible through other means than the S1–S2 time difference alone. As the electron swarm is drifted under the applied electric field, it spreads with a width proportional to $\sqrt{z}$ due to diffusion. This results in pulses for the recorded S2 signal which are wider in time for events that have drifted from larger *z*. Consequently, the study of the signal shapes in the S2 pulse can in principle also be used to determine the *z* position of an event. The NEXT program has characterized diffusion in xenon gas at various pressures and electric fields [8, 9]. At the 41 V/cm/bar operating point of NEXT-White, the longitudinal reduced diffusion constant is approximately $D_L=1000~\sqrt{\textrm{bar}}~{\upmu \text {m}}/\sqrt{\textrm{cm}}$ and the transverse reduced diffusion constant $D_T=3800~\sqrt{\textrm{bar}}~{\upmu \text {m}}/\sqrt{\textrm{cm}}$.

If achievable, this technique yields several advantages. One is that having redundant methodologies for determining event position can enable more cross checks, better position reconstruction, improved background rejection or selection efficiencies. For example, if a prospective event with matched S1 and S2 signals, is found to have an S2 width that is different than would be expected from diffusion given the time difference between the S1 and S2 signals, it can be rejected as having an incorrectly assigned S1, potentially through accidental coincidence. Furthermore, if a single S2 event is found accompanied by two potential S1 signals, it would traditionally be rejected. By using the diffusion information, the correct S1 signal can be identified, increasing the selection efficiency. This is likely to be an especially useful technique for ^83m^Kr calibration of large-scale future detectors [10], where pileup of events could otherwise become a limiting factor in detector calibration and hence energy resolution. Finally, this method could also allow for a xenon TPC to operate with limited access (or even without) to S1 information. Although noble element TPCs have proven highly scalable to date, advancing to new detector scales will present technical challenges. The light collection requirements for the small S1 signals are more severe than those for the larger S2 signals, the latter being amplified through electroluminescence. With multiple R &D pathways now being explored to realize future very large xenon TPCs [11–15], understanding the information content in each signal component is of significant interest.

In this paper, methods for identifying the position of an event in the drift (z) direction based on the signal width of the diffusion of the ionization electrons are demonstrated using the NEXT-White experiment. In Sect. 2, the detector and data set are briefly described. In Sect. 3, two methods using signal width from diffusion in order to determine the *z* position of point-like ^83m^Kr calibration events are developed, which employ analytical quantification of the shape of the waveform. In Sect. 4, a method is described for extracting the *z* position from events at higher energies where the more complex track topologies requires analysis via machine learning algorithms. In both cases, reconstruction of event *z* position with few-cm precision is demonstrated. Finally, Sect. 5 presents the conclusions.

## The NEXT-White and NEXT-100 detectors

NEXT (Neutrino Experiment with a Xenon TPC) is an experimental program aiming at the detection of $0 \nu \beta \beta$ decay in ^136^Xe, using successive generations of high pressure gaseous xenon electroluminescent time projection chambers (HPXe EL-TPCs) [16]. Small scale prototypes demonstrated the capability of the technology to achieve sub-$1\%$ FWHM energy resolution and to topologically identify signal-like events [17, 18], and this capability has since been tested underground with the larger ($\sim$5 kg of ^136^Xe at 10 bar) NEXT-White detector [19–22], at the Laboratorio Subterráneo de Canfranc (LSC) in Spain. NEXT-White has measured two neutrino [23] double beta decay and demonstrated the feasibility of neutrinoless [24] double beta decay searches based on event-by-event topological identification and a direct background subtraction between enriched and depleted xenon. In addition, NEXT-White served as a test-bed to inform the designs of future NEXT experiments including NEXT-100 [25] and ton-scale phases [10].

**Fig. 1:**
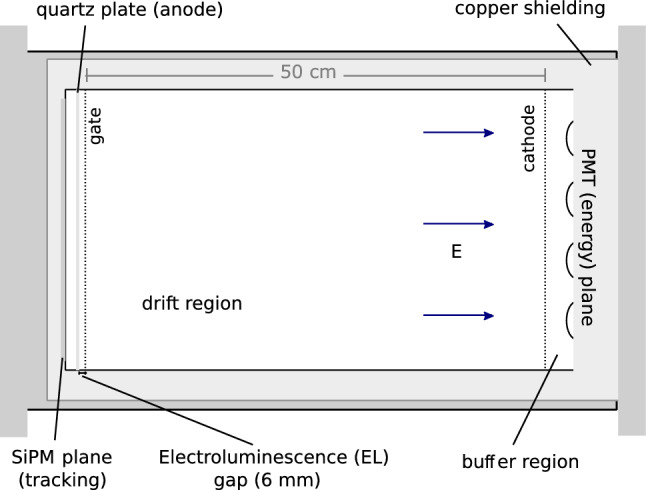
Schematic of the EL-based TPC developed by the NEXT collaboration for neutrinoless double beta decay searches in ^136^Xe, from [20]

The cylindrical NEXT-White TPC (shown schematically in Fig. 1) has a length of 53 cm and a diameter of 40 cm. The energy of each event is measured by twelve Hamamatsu R11410-10 photomultiplier tubes (PMTs) placed 130 mm from a transparent wire array cathode. The events are imaged by a 2D-array (10 mm pitch) of 1792 SensL C-Series, 1 mm^2^ silicon photomultipliers (SiPMs), placed a few mm behind an electroluminescence (EL) gap of 6 mm. The drift region has an electric field of 40 V cm^-1^ bar^-1^ and the EL region is defined by a stainless steel mesh and a grounded quartz plate coated with indium tin oxide (ITO) and tetraphenyl butadiene (TPB) thin films. More details on the NEXT-White detector can be found in Ref. [22].

**Fig. 2:**
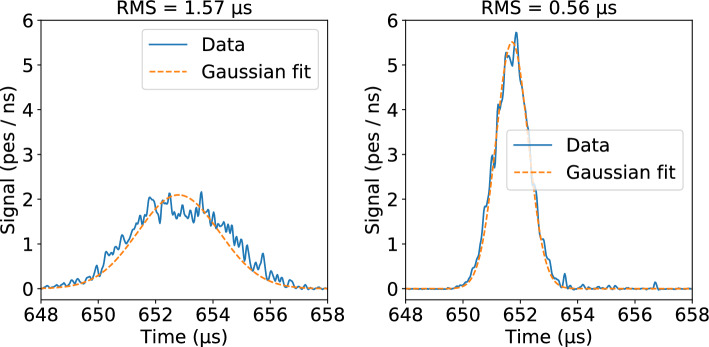
Two examples of ^83m^Kr events as a function of time, where signals from all 12 PMTs are summed, overlaid with Gaussian fit with width fixed to calculated RMS value. Time widths of events as measured by root mean squared indicated above the corresponding plots

The combination of the tracking information with the time of the event from the S1 signal (t_0_) provides the 3D (*x*, *y*, *z*) positions of events.

This information is typically required for fiducialization, to veto events occurring near the edges of the detector where background events are more likely, and to apply position-dependent corrections for electron attachment on impurities phenomena required to achieve the target energy resolutions of $\sim$1% FWHM as reported in [20]. For NEXT-White, continuous detector calibration and monitoring are conducted by introducing radioactive ^83m^Kr into the detector volume [26]. ^83m^Kr, a noble gas, decays primarily via low-energy electron capture (41.5 keV), resulting in the generation of point-like events uniformly distributed throughout the detector volume. This calibration procedure enables correction for spatial variations in the detector on a daily basis, as well as for the finite electron lifetime caused by the attachment of ionization electrons to impurities before their collection [26].

The coming phase of the NEXT program, NEXT-100 is presently under construction, and aims to demonstrate an ultra-low background search for $0\nu \beta \beta$ in high pressure xenon gas at the 100 kg scale [27]. The NEXT-100 TPC is approximately 1 m long and 1 m in diameter, scaling up linear dimensions of NEXT-White by a factor of two.

## Reconstruction of the *z* position of low-energy ^83m^Kr electron captures using diffusion

^83m^Kr has proven central to achieving position-dependent calibration of the NEXT detector, both for nonuniformities in (*x*, *y*) (the plane perpendicular to the drift direction), and for variations in *z* (the drift direction) due to electron attachment. ^83m^Kr decay events are excellent candidates to study the position reconstruction from diffusion, as they are close to point sources at production. This indicates that the width resulting from the diffusion of the ionization electrons can be quantified by examining the shape of the electron cloud detected. The S1 signals produced by ^83m^Kr events are the lowest energy signals used in NEXT, and their detection could thus be among the more challenging aspects of future large detector design.

The ionization cloud diffuses in both the transverse and the longitudinal directions during drift. A Gaussian electron cloud with longitudinal width *d* traveling at velocity *v* will produce an approximately Gaussian pulse of light with width in time of approximately *d*/*v* when entering the EL gap, with a small correction from the time it takes to cross the gap. Non-Gaussian corrections to the pulse shape were studied in Ref. [9] and found to be negligible. In contrast, the transverse width impacts the distribution of light across the SiPMs of the tracking plane, and its precision is limited by the 1 cm SiPM spacing. For this reason, the optimal diffusion-based measure of *z* for krypton events is extracted from longitudinal diffusion only. The width of the pulse in time, also referred to as the “(longitudinal) event width”, is measured in terms of the root mean squared (RMS) of the pulse. According to the diffusion equation, the RMS^2^ is expected to increase linearly with drift distance (*z* position). Longitudinal diffusion in the NEXT-White detector has been previously quantified to have an RMS spread of $0.3\, mm / \sqrt{cm }$ [28].

**Fig. 3:**
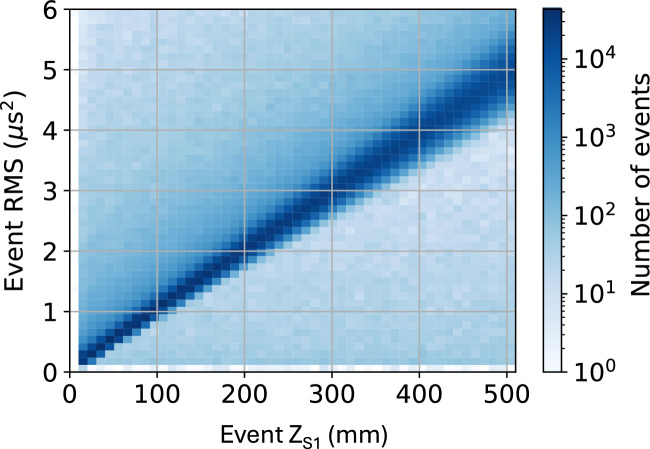
The square of the longitudinal root mean squared (RMS^2^) width of ^83m^Kr events as a function of the *z* position obtained from the S1 signal in the NEXT-White detector. A clear linear relationship between the two is observed, as expected

The study presented here uses 7 million ^83m^Kr events taken over the course of a single day in NEXT-White. Two examples of ^83m^Kr event pulses as a function of time can be seen in Fig. 2. In Fig. 3, the distribution of event widths (in RMS^2^) as a function of *z* position (determined from S1) is shown. The linear increase of RMS^2^ with *z*, as anticipated from diffusion, can be observed.

**Fig. 4:**
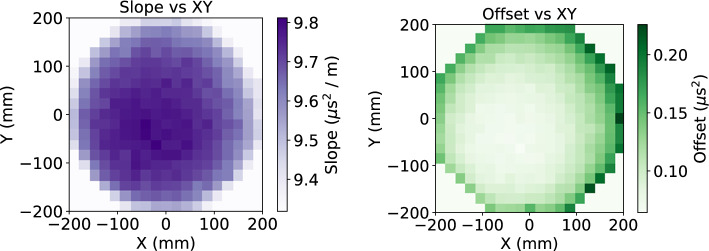
Linear fit parameters of ^83m^Kr event RMS^2^ as a function of *z* (from S1) in NEXT-White for different *x* and *y* locations. Left: Slope of the linear fit, corresponding to diffusion. Right: Offset of the linear fit, corresponding to typical width of ^83m^Kr event at *z* $= 0$ mm. A clear dependence of both parameters with *x* and *y* is seen

This measured linear relationship allows extraction of the *z* position of a ^83m^Kr event given the RMS^2^ of the pulse, named $z_{\textrm{RMS}}$. The offset corresponds to the width of a typical ^83m^Kr event which occurs exactly at the EL gap, where there is almost no diffusion, while the slope corresponds to the impact of diffusion along *z*. These parameters are extracted as a function of *x* and *y* position. To provide $z_{\textrm{RMS}}$ positions for all (*x*,*y*) locations, the geometry is sub-divided into $19 \times 19$ (*x*, *y*) bins, each $10.5 \times 10.5~$mm^2^. For each bin, a linear fit to the relationship between RMS^2^ and *z* is performed and the values of slope and offset are extracted. The observed spatial variation of the fitted parameters is shown in Fig. 4. A plausible explanation for the small variations in the offset parameter are position dependence in the width of the EL gap. Variations in the fitted slope appear to reflect differences in the extracted diffusion coefficient. This could be a consequence of non-uniformity in the electric fields near the detector boundary. These offset variations are small, with a standard deviation of 1.3%.

**Fig. 5:**
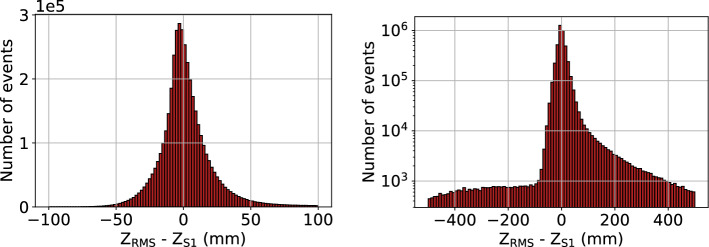
Differences between *z* positions determined by RMS ($z_{\textrm{RMS}}$) and determined from S1 ($z_{\textrm{S1}}$) for ^83m^Kr events in NEXT-White, shown in linear (left) and log (right) scales

**Fig. 6:**
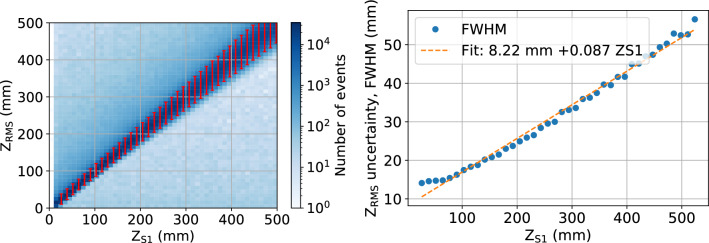
Left: *z* position estimated from width ($z_{\textrm{RMS}}$) in function of the *z* position assigned from S1 ($z_{\textrm{S1}}$) for ^83m^Kr events in NEXT-White. Red uncertainties, representing FWHM of $z_{\textrm{RMS}}$ in a given $z_{\textrm{S1}}$ range, are overlaid. Right: FWHM of $z_{\textrm{RMS}}$ as a function of $z_{\textrm{S1}}$ in NEXT-White, with a linear fit, understood as the increase in uncertainty of the $z_{\textrm{RMS}}$ with $z_{\textrm{S1}}$

The $z_{\textrm{RMS}}$ position values obtained from the linear fit to the RMS^2^ distributions as described above can be compared to the *z* positions obtained from the S1 signal ($z_{\textrm{S1}}$) in Fig. 5. $z_{\textrm{RMS}}$ is seen to have a small overall bias compared to $z_{\textrm{S1}}$, with an overall median shift of $z_{\textrm{RMS}}-z_{\textrm{S1}}=-0.20$ mm. The error $|z_{\textrm{RMS}} - z_{\textrm{S1}}|$ averaged over the whole detector is 9.4 mm, indicating most events are estimated using the RMS method as within 1 cm of the position assigned using the S1 signal. Long and non-Gaussian tails on the positive end of the distribution of $z_{\textrm{RMS}}-z_{\textrm{S1}}$ indicate a population of events much wider (RMS much larger) than would be predicted from S1. This could be due to events with incorrectly assigned S1 pulses, for example in a case where part of the S2 signal is misinterpreted as an S1. The distribution of $z_{\textrm{RMS}}$ as a function of $z_{\textrm{S1}}$ is shown in Fig. 6. The distribution is overlaid with error bars indicating the FWHM spread in the distribution of $z_{\textrm{RMS}}$ values in fixed $z_{\textrm{S1}}$ bins. These indicate the spread in assigned $z_{\textrm{RMS}}$ values given a fixed, known $z_{\textrm{S1}}$, and are interpreted as the uncertainty in the extraction of $z_{\textrm{RMS}}$. The uncertainty is seen to increase linearly with $z_{\textrm{S1}}$ at a rate of $87 \, mm /m$, as estimated from the right panel of Fig. 6.

**Fig. 7:**
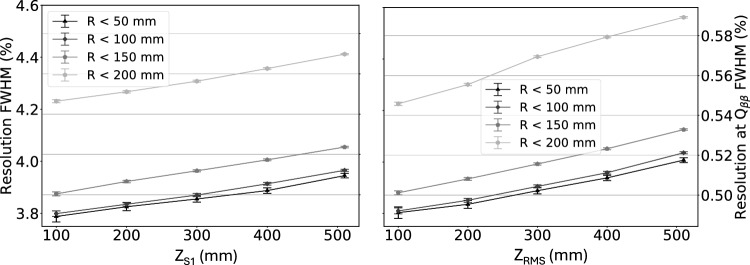
Energy resolution for ^83m^Kr events as a function of $z_{\textrm{S1}}$ in NEXT-White, for regions of varying maximum distance from central axis *R*. Left axis indicates resolution in FWHM / 41.5 keV for both data sets, and right axis is matched to left axis to indicate resolution extrapolated to Q_ββ_ for both data sets. Volumes are overlapping, with $z_{\textrm{S1}} = 300~$mm including all points with $z \le 300~$mm, for example. Left: Energy resolution calculated using $z_{\textrm{S1}}$. Right: Energy resolution calculated using z_RMS_

One of the key goals of using ^83m^Kr in NEXT is energy resolution calibration. Variability in the detected brightness of ^83m^Kr events over the detector is used to generate the detector response correction that is applied to higher energy events. Any imprecision in the *z* reconstruction thus implies an imprecision in energy calibration. Energy resolution for ^83m^Kr events is defined as the energy peak percent FWHM, and is measured for both $z_{\textrm{RMS}}$ and $z_{\textrm{S1}}$ as a function of position by subdividing the detector into several (overlapping) volumes of increasing maximum event radius (*r*) and *z*-position. The resolution comparison can be seen in Fig. 7 for NEXT-White data, where a slight degradation in resolution is observed for $z_{\textrm{RMS}}$ as compared to $z_{\textrm{S1}}$. This extrapolates to a change by around $0.01\%$ at $Q_{\beta \beta }$. Such a difference is sure to be negligible when determining sensitivity to neutrinoless double beta decay.

In order to analyze the applicability of the aforementioned method to larger detectors, 1 million ^83m^Kr events were generated using a Monte Carlo simulation in the NEXT-100 detector, in a configuration resembling as close as possible the anticipated running configuration of the detector.

**Fig. 8:**
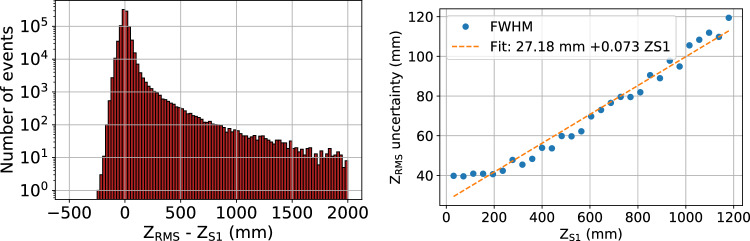
NEXT-100 simulation. Left: Differences between *z* position determined by RMS ($z_{\textrm{RMS}}$) and determined from S1 ($z_{\textrm{S1}}$) for ^83m^Kr events shown in log scale. Right: FWHM of $z_{\textrm{RMS}}$ as a function of $z_{\textrm{S1}}$ with a linear fit, understood as the increase in uncertainty of the $z_{\textrm{RMS}}$ with $z_{\textrm{S1}}$

The distribution of differences between the *z* positions assigned from diffusion ($z_{\textrm{RMS}}$), and from S1 ($z_{\textrm{S1}}$) in NEXT-100 is shown in the left of Fig. 8. The long non-Gaussian tails are comparable to those observed in NEXT-White, with median difference of $-$0.28 mm (compared to $-$0.20 mm in NEXT-White), again indicating a lack of significant bias in a particular direction. The error $|z_{\textrm{RMS}} - z_{\textrm{S1}}|$ averaged over the whole detector is 16.1 mm, somewhat larger than in NEXT-White. The energy resolution obtained with z_RMS_ is around $0.01\%$ worse in each volume than that achievable with z_S1_, comparable to what was seen for NEXT-White. The increasing uncertainty as a function of *z* thus translates to only a minuscule degradation of the energy resolution at the Q_ββ_ value. A similar linear relationship between uncertainty of $z_{\textrm{RMS}}$ assignment as a function of $z_{\textrm{S1}}$ can be seen in the right of Fig. 8.

**Fig. 9:**
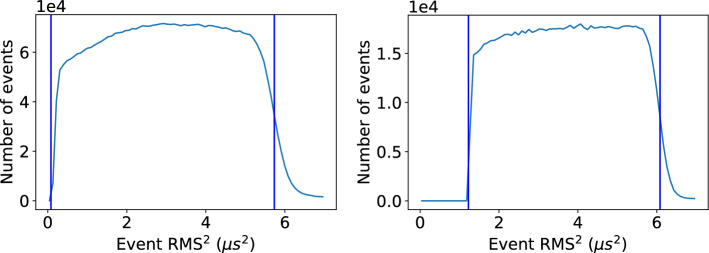
Distribution of mean squared (RMS^2^) widths of ^83m^Kr events, with boundary lines indicating corresponding minimum and maximum *z* values of the detector as determined from the distributions as described in the text. Left: NEXT-White data. Right: NEXT-100 simulation

It is notable that the described method for assigning the positions of ^83m^Kr events via diffusion did still rely on the use of S1 information indirectly, in order to build the calibration distributions of RMS^2^ as a function of $z_{\textrm{S1}}$. In a detector which would not have the S1 information, this would not be possible. Thus an alternative method must be used to calibrate the conversion between pulse width and *z*. Because RMS^2^ varies linearly with *z* position, a known RMS^2^ value at $z = 0$ mm and the maximal drift distance $z = z_{\textrm{max}}$ is sufficient to accomplish this tuning. The distribution of observed values for RMS^2^ shows a sharp rising edge for small values, but a long falling tail at maximal diffusion. Nevertheless, in both simulation and data it was found that $z_{\text {max}}$ corresponds closely to the right half-max of the RMS^2^ distribution. This is shown for NEXT-White data and NEXT-100 Monte Carlo in Fig. 9. That the same method works for both data and simulation indicates that this “boundary method” is a reasonable and robust way of establishing the mean diffused pulse widths corresponding to the detector extrema without the need for S1-based tuning.

**Fig. 10:**
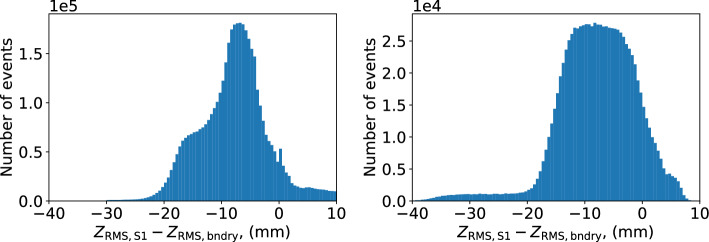
Distribution of differences between position of ^83m^Kr events as assigned using the linear correlation between RMS^2^ as a function of *z* from *S*1 ($z_{\textrm{RMS,S1}})$ and as assigned purely referencing the cutoffs of the RMS^2^ distribution and the known detector boundaries in *z* ($z_\mathrm {{RMS,bndry}}$), as described in the text. Left: NEXT-White data. Right: NEXT-100 simulation

Figure 10 compares the *z* position obtained with a diffusion curve calibrated using S1, and one calibrated using the boundary method for NEXT-White data and NEXT-100 simulation. The distributions have some qualitative differences, although in both cases errors tend to be slightly negative, with the boundary method assigning events as being slightly deeper (higher *z*) than the S1-referenced method. Errors are generally less than 20 mm in magnitude in either case. This level of imprecision is not expected to have any significant effect on key detector performance metrics, given the expectation of free electron lifetimes greater than 5 ms, which correspond to 4500 mm at the NEXT-White drift field.

## Reconstruction of the *z* position of $E>1.5$ MeV radiogenic electrons using diffusion

We now turn attention to measuring the *z* position of longer tracks from high energy electrons based on their diffusion. Extraction of the *z* position of higher energy radiogenic events via diffusion is a more complex task than for the point-like deposits of ^83m^Kr. The events of interest, including photoelectrons and Compton electrons from gamma rays, as well as the two-electron signatures of either neutrinoless or two-neutrino double beta decays, present long, tangled topologies. The precise shape of the track will depend on both its local 3D structure and upon diffusion and the electroluminescent region response time profile. To extract the spread from diffusion while accounting for the structure of the track in 3D space thus requires an analysis of the whole topology rather than a direct quantification of the S2 pulse shapes. To this end, a neural network based approach was developed.

Deep neural networks have been employed for NEXT topological event reconstruction to distinguish between one-electron signatures of background events and two-electron double beta decay signatures. A method was first proven in [29], and honed in [30] to achieve substantial performance improvements in event classification and background rejection techniques beyond traditional approaches. Those works use the double escape peak of ^208^Tl with energy of 1.6 MeV, as a monoenergetic calibration line of two-electron events, and use a network trained on Monte Carlo events to select the two-electron “signals” over one-electron “backgrounds” from the local Compton continua from various higher energy gamma-ray lines. Performance of the network was assessed based on how well the calibration peak at 1.6 MeV was extracted from backgrounds. This metric mirrors the requirement of distinguishing $0\nu \beta \beta$ events from ^214^Bi Compton events and ^208^Tl photoelectrons around the Q-value for $0\nu \beta \beta$ at 2.4 MeV.

**Fig. 11:**
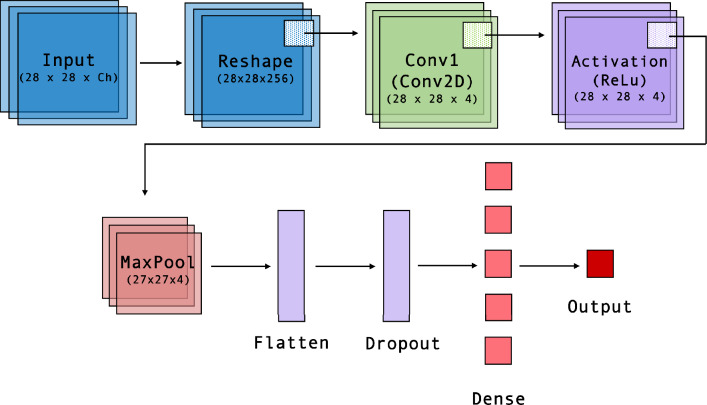
Network architecture for *XY* plane configuration. For all three planes, *XY*, *YZ*, and *XZ* a sequential model is constructed. A permute layer is added to models *YZ* and *XZ* for dimensional order. Key features are extracted from the Input to the MaxPool layers, and classification based on these features occurs from the Flatten to Output layers

Making classification decisions about complex tracks using information about the full 3D image is a natural application for deep neural networks. For the application described here, however, a different network structure appeared optimal. Whereas topology is a global decision about the track shape, the extraction of information on diffusion is a spatially localized process, and many local measurements may be expected to reinforce each other. This local information should be accessed while avoiding the possibility of over-training on complex track features. Thus the chosen network architecture is 1) convolutional, to measure features of local track regions; 2) shallow, to avoid encoding more than the simplest, local features into the classifier; and 3) trained with significant information dropout layers to avoid over-training on event topology details.

Both 2D and 3D convolutional approaches were assessed. Marginally better performance was achieved by utilizing three independent 2D convolutional networks. These networks are applied layer-by-layer to the event, and their outputs are combined in a single densely connected layer. Finally, the measured *z* position, representing the barycenter of the event, is communicated to an output neuron. This improved performance of 2D over 3D networks is attributed to the larger number of extra free parameters in the 3D network, which ultimately provides slightly more of a training burden than an advantage given that the diffusion process acts essentially independently in each orthogonal direction.

**Fig. 12:**
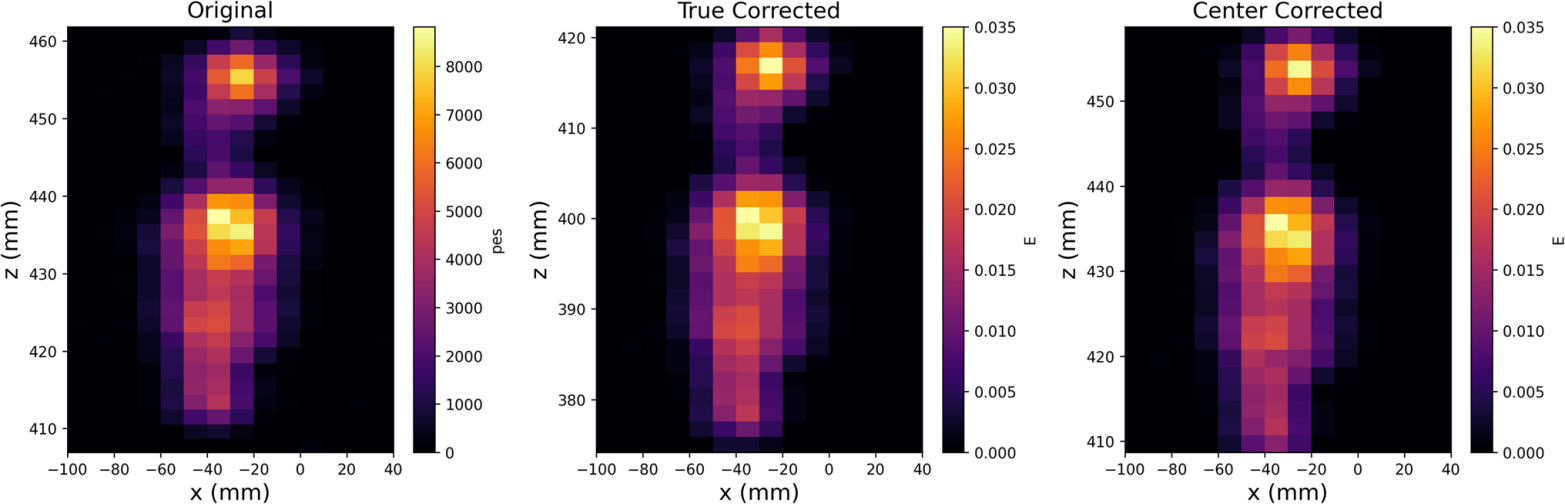
Representation of the original uncalibrated event (left), the event after S1 calibration (middle), and the event post-diffusion calibration (right), projected onto the x-z plane. The effects of these corrections are notably rather small

The three projections of the NEXT drift volume are inequivalent due to different event discretization scales in the transverse and longitudinal directions, with 10 mm SiPM pitch spacing transversely and 3 mm digitization distance longitudinally. There are also different longitudinal and transverse diffusion constants, and distinct mechanisms contributing to event spreading during detection. Longitudinally, the event is broadened by the EL crossing time of 2 $\upmu$s. In contrast, transversely, event spreading occurs by a non-Gaussian point-spread function associated with distribution of the VUV photons on the wavelength shifting plate. For these reasons the network acting in the purely transverse *XY* plane has different optimal parameters than the two acting in the longitudinal-transverse *XZ* and *YZ* planes, and they are trained independently.

The network architecture follows a sequential model where a series of layers are applied. Each model is composed of a 2D convolutional layer along with an activation layer using the rectified linear unit function (relu), followed by a max pooling operation layer [31]. The model then uses a flatten and dropout layer to prevent over fitting during training. Two consecutive activation layers applying the relu function are accompanied by their own dense connected layer. The model is compiled for training using a mean of squares loss function between the true and predicted values. For each individual plane, a reshape and permute layer is incorporated before the 2D convolutional layer according to its *x*, *y* and *z* input dimension. A visual representation of the *XY* network architecture is shown in Fig. 11.

**Fig. 13:**
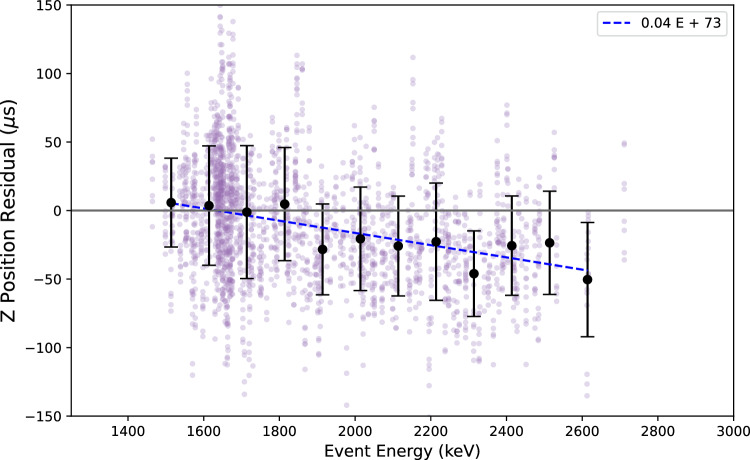
Measurement of energy bias on *z* residuals as a function of center energy. The best fit line in blue is used to correct events based on their center energy after initial *z*-placement by the neural network

The network is trained using real NEXT-White tracks containing one S1 pulse and one S2 pulse. To produce S1-stripped data, raw events were artificially moved to have their mean *z* positions at the center of the fiducial volume. The raw hit charges were calibrated with krypton maps derived from the diffusion-only measurements of Sect. 3, and applied to the event at this new artificial position rather than its S1-reconstructed *z* location. This leads to a partially calibrated “center-corrected” event, which has an approximately reconstructed energy that we denote its “center energy”. This procedure implements the two calibrations that are possible with no S1 information: 1) correction of purely *XY*-dependent effects such as differential SiPM response, EL or WLS plate non-uniformity, and 2) the small adjustment to the *Z* shape of the event from the electron lifetime correction, with longer-drifting electrons within the event being slightly more attenuated than shorter-drifting ones. The overall event attenuation correction that typically uses *z* from S1 is not applied. Orthogonal subsets of such events are used as both training and test samples. Figure 12 shows an event before calibration, after S1 correction, and after being center-corrected.

A data driven approach was developed to train the network that extracts *z* position information from center corrected data. The network was trained to learn the S1-derived *z* position for each center corrected event. The training is run for 30 epochs for 3 uninterrupted passes. Training and validation were performed with the subset of fiducialized events with reconstructed energy above $E\ge 1.5$ MeV. These longer, most tangled events are not only the most challenging to extract the diffusion scale from, but also of the most interest for NEXT analyses including $0\nu \beta \beta$ searches, double-escape peak calibration of the NEXT topological signature, and calibration of the detector energy resolution using for example the ^208^Tl 2.6 MeV photo-peak.

**Fig. 14:**
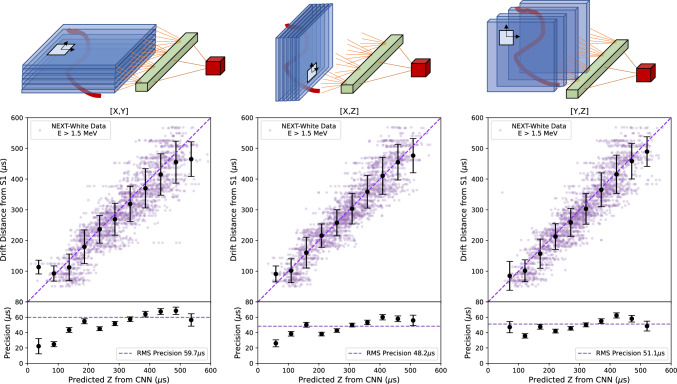
Top: Cartoon illustrating the projections for each of the convolutional network outputs (left: *XY*, middle: *XZ*, right: *YZ*). In the 2D convolutional layers with filters, the focus is on capturing the diffusion effect locally around the track signature. This information is then combined onto the output node through a dense layer, as depicted in Fig. 11. Bottom: Correlation between drift distance from S1 and predicted *z* from CNN for each projection. The purple points represent the barycenter of NEXT-White data events, while the black points represent the mean for each binned slice, with error bars indicating the standard deviation. The purple diagonal dashed line illustrates an ideal prediction, for reference. The lower panels display precision, represented by the size of the standard deviation, with the RMS precision shown by the dashed horizontal line

A total of 3,600 events passed these selection cuts. In order to maximize the statistical power of the training set each event was subjected to eight symmetry transformations in the transverse plane: this includes every combination of two possible mirroring operations and four rotations. The augmented training set is thus a factor of eight times larger than the original dataset, which improves the precision of the final network since its training is statistically limited. This method can be used in the transverse plane but not in either of the longitudinal ones, since the front and back end of the track in the drift direction are not equivalent due to dissimilar diffusion scales at front and back. This symmetrization of the network training is further exploited by averaging the result of operating the network on each of the eight symmetry transformations of each test event to provide the final estimate of its *z* position.

The total energy for each event is normalized to a constant before either training or validation, so that the network is forced to extract the diffusion width from spatial information rather than using information on event brightness to estimate *z*. The location of the event is taken to be its barycenter, the summation of the hits *z* times its energy divided by the events total energy. After training, a small bias in the *z* reconstruction as a function of energy was observed, and this is corrected with a linear function derived from the data, as shown in Fig. 13. This correction is typically far smaller than the physical size of the event and makes only a marginal difference to the final average precision over the dataset.

**Fig. 15:**
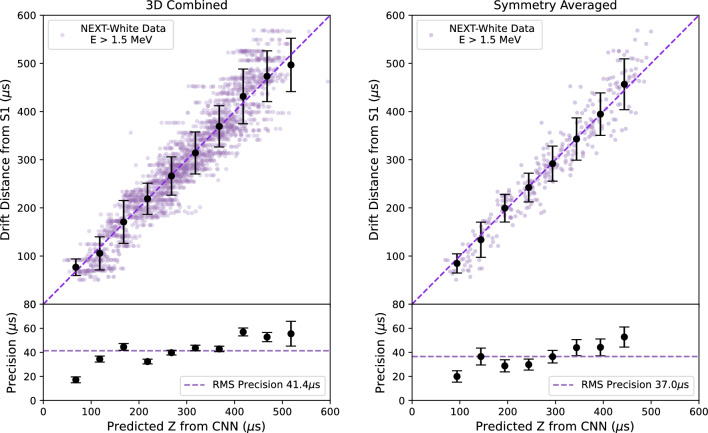
Correlation between drift distance from S1 and predicted *z* from CNN. The purple points represent the barycenter of NEXT-White data events, while the black points represent the mean for each binned slice, with error bars indicating the standard deviation. The dashed lines have the same interpretation as in Fig. 14. Left: The average of all 3 convolutional network configurations. Right: Average obtained from the eight symmetry transformations to the events from the plot on the left

**Fig. 16:**
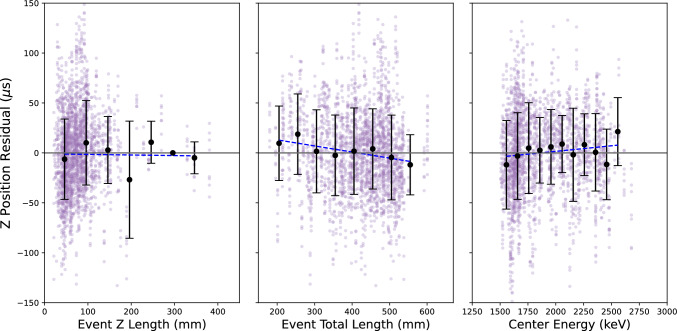
Tests for biases in the event *z* precision as a function of event characteristic shape and energy: (left) *z*-extent, (middle) total event length, and (right) true event energy. The purple points indicate NEXT-White events after CNN application and center energy correction. The black points represent the mean for each binned slice, with error bars indicating the standard deviation. The blue dashed line represents the fit of all the black points

The validation set is composed of 8% of the data reserved from the training set to assess the network performance. The average *z*-location precision from each plane for the data driven network are *XY*: 59.7 $\upmu$s, *XZ*: 48.2 $\upmu$s and *YZ*: 51.1 $\upmu$s, as demonstrated in Fig. 14. The final prediction of the network was determined by an averaged sum of outputs from the *XY*, *YZ*, and *XZ* networks. By taking the averaged sum, the precision further improved to 41.4 $\upmu$s as seen on Fig. 15. These can be converted into distance scales by multiplying by the drift velocity, approximately 0.91 mm/$\upmu$s. A further small improvement was obtained by exploiting the symmetry properties of the detector previously described. The *z* precision after this procedure was found to improved to 37.0 $\upmu$s. The final network performance is shown for the validation sample in Fig. 15. A linear relationship can be seen between the network predicted *z* and drift distance from S1.

The performance of the diffusion-based *z* reconstruction protocol was assessed as a function of event center energy, total length and length in the *z* direction to test for possible biases. No strong dependencies of precision upon on these variables were observed. These tests are shown in Fig. 16.

We thus conclude that events can be reconstructed in 3D space using diffusion to measure *z*, with a demonstrated precision of approximately 37 $\upmu$s, or 33.6 mm. This precision is small relative to the measured electron lifetime of between 5 ms and 14 ms in NEXT-White [1], suggesting the method is sufficiently precise to calorimetrically correct event energies without *z*-positioning becoming a limiting factor for energy resolution in a diffusion-based reconstruction chain. Using the 7 ms lifetime of the run considered in this study, the implied energy resolution at Q_ββ_ would be modified from the 1% FWHM [20] value measured in NEXT-White to 1.1% FWHM, adding the uncertainty introduced by *z* positioning in quadrature. The method is also precise enough to reject false S1–S2 coincidences, with potentially improvements for signal selection efficiency or background rejection factors in future double beta decay analyses, and to fiducialize events to reject cathode-originated radiogenic backgrounds.

## Conclusions

In this article, several methods of determining event *z* position using diffusion in a pure xenon time projection chamber were demonstrated. Fitting the pulse shapes of ^83m^Kr S2 signals yields uncertainties that are generally less than $25 \text { mm}$ in NEXT-White. Using Monte Carlo simulation, it is shown that this method can be extended to a larger detector such as NEXT-100, with similarly negligible degradation to energy resolution. This method was used to generate calibration maps using Kr that can be applied to events with energy > 1.5 MeV, even in the case where S1 information is absent in their generation.

A convolutional neural network based approach has been demonstrated to reconstruct the *z* position for higher energy ($E>1.5$ MeV) events via diffusion. A data driven training method was used to construct a network capable of estimating the *z* position of detected events based on their diffusion. The final *z* precision was found to be 33.6 mm, by averaging the weighted predictions of the *XY*, *YZ* and *XZ* networks over symmetry configurations. This is far smaller than the measured electron lifetime, suggesting promise as a method for longitudinal event reconstruction without S1.

This method can serve useful purposes, even in detectors which have full access to S1. As one example, it enables a new event quality check to reject events with inaccurately determined characteristics, such as in cases of event pileup where S1 to S2 association becomes ambiguous. The cross-check provided by an independent *z* position estimate from diffusion enables the rejection of events with improper S1-to-S2 associations. This can provide higher event selection efficiencies and better background rejection capabilities, as well as allowing for higher calibration event rates in future large detectors. The results presented also indicate the potential to use diffusion in a pure xenon time projection chamber to reconstruct the *z* position of events even if no S1 signals are available.

## Data Availability

Data will be made available on reasonable request. [Author’s comment: The datasets generated during and/or analysed during the current study are available from the corresponding author on reasonable request.]

## Code Availability Statement

Code/software will be made available on reasonable request. [Author’s comment: The code/software generated during and/or analysed during the current study is available from the corresponding author on reasonable request.]
